# Tools for Detecting Ageing in People with Autism Spectrum Disorder: A Scoping Review

**DOI:** 10.3390/healthcare13202640

**Published:** 2025-10-20

**Authors:** Maider Ugartemendia-Yerobi, Beatriz Pereda-Goikoetxea, Maria Isabel Trespaderne, Jaione Lacalle

**Affiliations:** Faculty of Medicine and Nursing, Department of Nursing II, University of the Basque Country, 20014 Donostia-San Sebastian, Spain; beatriz.pereda@ehu.eus (B.P.-G.);

**Keywords:** aging, Autism Spectrum Disorder, assessment, frail elderly

## Abstract

Background: People with Autism Spectrum Disorder (ASD) require a customised, multidisciplinary plan throughout their lifetime to support optimal health. The purpose of this scoping review was to synthesise research on the main scales used to detect signs of ageing in people with ASD. Methods: Eligible papers published between January 2003 and August 2025 were identified through searches of PubMed, PsycInfo, Scopus, Web of Science, NICE and Cochrane databases. The assessment was performed using the Joanna Briggs Institute critical appraisal and extraction checklist. Of the 820 papers reviewed, 24 were found to meet the established criteria. Results: Based on the evidence collected, 57 tools focusing on specific domains within the Comprehensive Geriatric Assessment were identified: 19 addressed the functional domain, 18 the mental, 6 the biomedical, 1 the social, 2 related to frailty, 1 to fall risk, and 10 to quality of life. Conclusions: This review highlights the need to obtain a ‘multi-domain’ tool for the detection of ageing in autistic people, which would facilitate the development of a Comprehensive Geriatric Assessment that makes planning customised care possible.

## 1. Introduction

It is expected that due to population ageing and the increase in chronic diseases, by 2050, the global population of people over 60 years of age will more than double and reach 2100 million [1]. The World Report on Ageing and Health acknowledges that increasing longevity depends to a large extent on healthy ageing, that is, on developing and maintaining, even at an advanced age, the functional capacity necessary for well-being [2].

Although the likelihood of living longer constitutes an important collective achievement, a great inequality in longevity persists depending on the social and economic group to which one belongs. Similarly, authors such as Rowe and Kahn [3] suggest that people with disabilities may experience ‘unsuccessful ageing’; however, rather than implying their exclusion, this highlights the need for their prioritisation in initiatives aimed at promoting healthy ageing. In this sense, alongside social and economic factors, neurotype should also be considered, as autistic individuals face specific barriers that contribute to increased morbidity and mortality [4].

Autism Spectrum Disorder (ASD) is a neurodevelopmental disorder that affects both children and adults [5]. Currently, the DSM-5 establishes a new classification system based on a single spectrum that better acknowledges the diversity of ASD, characterised by an atypical social and communication style and restricted interests and/or repetitive behaviours [6], areas referred to as core symptoms [7]. It should also be noted that there is a high comorbidity between ASD and intellectual disability (ID) [8], affecting approximately 50% of autistic people [9]. 

Scientific evidence suggests that people with autism begin to experience biopsychosocial changes from the age of 40 onwards. This early deterioration is associated with an increased risk of premature mortality [10]. Compared to the general population, people with ASD experience poorer outcomes at all stages of the life cycle [11], with a dementia prevalence of 1.9% in men and 3.2% in women [12], and a higher incidence of Parkinson’s disease [10]. Similarly, evidence indicates that individuals with intellectual disabilities begin to experience a decline in their quality of life from approximately 45 to 50 years of age [13].

People with ASD need a customised, lifelong multidisciplinary plan that undergoes constant review and monitoring to support their full potential, social integration, and quality of life [14]. In this context, the Comprehensive Geriatric Assessment (CGA), a central tool in geriatrics, is an interdisciplinary, dynamic, and multidimensional process that assesses both the capacities and needs of an individual while identifying potential issues. It also facilitates the multidisciplinary diagnostic process and enables care planning focused on enhancing quality of life and maximising overall health for older adults [15,16]. Understanding the progression of age-related changes in people with ASD will thus aid in planning necessary supports for this stage of life [17,18].

Traditionally, tools developed for individuals with intellectual disabilities have been used to assess people with ASD, due to the high comorbidity between both conditions. In this regard, several authors report a notable lack of research on the ageing process and autism [7,19]. Therefore, the objective of this review is to identify the main scales that facilitate the detection of ageing in people with ASD, taking into consideration tools developed both for people with ASD and for those with intellectual disabilities.

## 2. Methods

### 2.1. Design and Research Question

A scoping review was conducted and the process was reported according to the Preferred Reporting Items for Systematic reviews and Meta-Analyses extension for Scoping Reviews (PRISMA-ScR) [20]. To examine existing scientific evidence, we framed our research question based on the PICO framework [21]—Population: People with ASD; Intervention: Assessment tools; Comparator: Not applicable; and Outcome: Identification and assessment of ageing process. Therefore, the following research question was formulated: Which tools for the detection of ageing in people with ASD exist?

### 2.2. Search Strategy

The search was conducted in MEDLINE (PubMed), PsycInfo, Scopus, Web of Science (WOS), the National Institute for Health and Care Excellence (NICE), and Cochrane from 1 January 2003 to 15 August 2025, with no language restrictions. Results were updated on 27 August 2025. Based on the terms “Autism Spectrum Disorder”, “Intellectual Disability”, “Aging”, “Frailty”, “Frail Elderly”, and the free term “assessment”, we used the following search command: (((“Autism Spectrum Disorder”[Mesh]) OR (“Intellectual Disability”[Mesh])) AND (((“Aging”[Mesh]) OR (“Frailty”[Mesh])) OR (“Frail Elderly”[Mesh]))) AND (assessment). The search was completed with additional papers obtained through the snowball method with backward citation tracking [22,23]. The search strategies used in various databases are detailed in Supplementary File S1.

### 2.3. Inclusion and Exclusion Criteria

The scope of this review was established via inclusion and exclusion criteria (Table 1).

### 2.4. Study Selection

The initial search identified 820 papers (PubMed: 187, PsycInfo: 16, Scopus: 299, WOS: 197, NICE: 85, and Cochrane: 36). After removing duplicates, two authors (M.U.-Y. and B.P.-G.) assessed the eligibility of 735 papers (including the 9 identified through snowball sampling). They independently examined the titles and abstracts, eliminating 634 papers for irrelevance. The remaining 62 papers were examined in full text.

**Figure 1:**
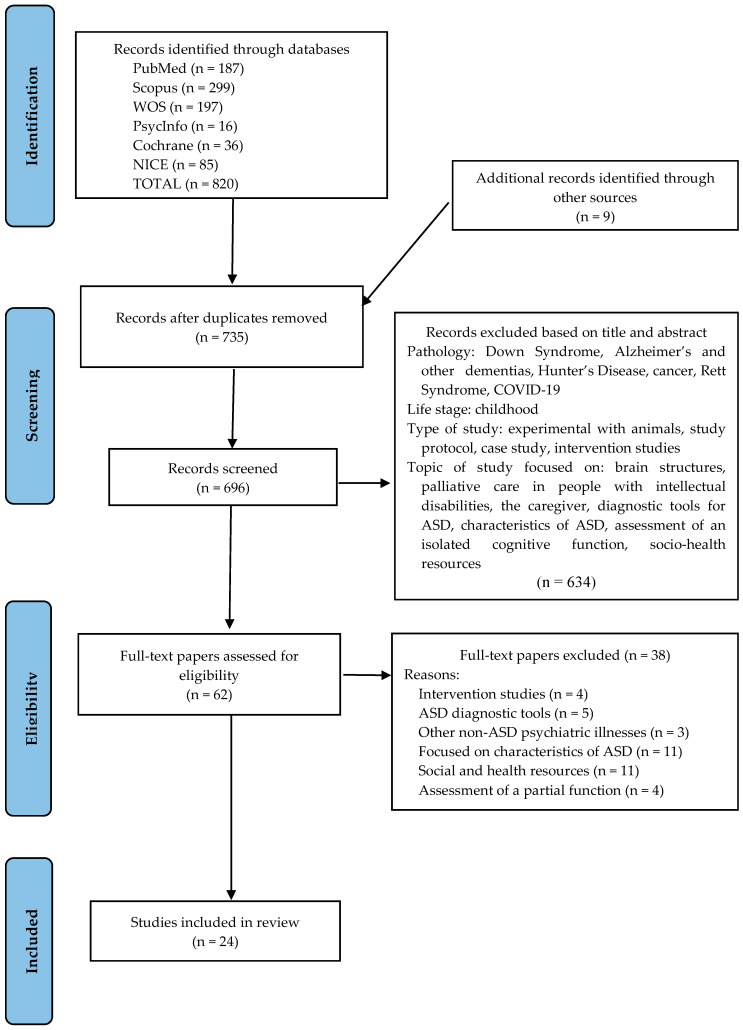
Prisma Flow Diagram of Study Selection. From: [20].

Two other investigators (J.L. and M.I.T.) resolved disagreements during the study selection process through discussion until consensus was reached. As reported in Figure 1, a total of 24 papers met the eligibility criteria, while a list of the 38 excluded studies is provided in Supplementary File S2. 

### 2.5. Quality Appraisal

The Joanna Briggs Institute (JBI) critical appraisal and extraction checklist was used to review and assess the rigor of the included papers [24]. Two researchers (M.U.-Y. and B.P.-G.) independently reviewed the papers and extracted the data before querying the identified common categories. The main issues were debated until a consensus was reached, and any disagreements between these two researchers were reviewed by two other researchers (J.L.P. and I.T.B.) to maintain the rigor of the review process (Supplementary File S3). 

### 2.6. Data Extraction

Prior to data extraction, the protocol for this study was registered in PROSPERO (ID: CRD42024571799). Two authors (M.U.-Y. and B.P.-G.) used a narrative approach to data extraction using a structured template. The data extracted from the selected papers were as follows: author, country, year of publication, design, tools used, participant characteristics, and key research findings (Table 2).

## 3. Results

Traditionally, CGA has been aimed at identifying four main domains: biomedical, functional, social, and mental. CGA comprises other domains such as frailty, nutritional status, falls, sarcopenia, and quality of life [15]. 

**Figure 2:**
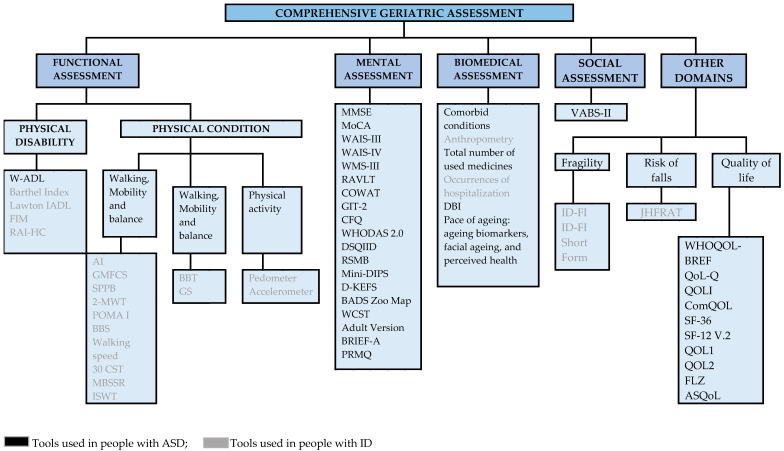
Comprehensive geriatric assessment tools identified in the scoping review.

The CGA tools obtained in the scoping review are shown in Figure 2. A total of 57 tools have been identified, of which 34 have been used in people with ASD and 23 in people with ID. Specifically, 19 tools correspond to the functional domain (1 ASD, 18 ID), 18 to the mental domain (18 ASD), 6 to the biomedical domain (4 ASD, 2 ID), 1 to the social domain (1 ASD), 2 to assess frailty (2 ID), 1 for the risk of falls (1 ID), and 10 for quality of life (10 ASD). Supplementary File S4 presents the availability and key psychometric properties of these scales.

### 3.1. Tools Used in People with ID Functional Assessment Tools

Functional assessment includes two major components: physical disability and functional limitation [15]. It ranges from basic motor tasks and self-care abilities to exercising and maintaining independence in various environments.

The tools for assessing physical disability are detailed below:

- Waisman Activities of Daily Living Scale (W-ADL), adapted for adults with developmental disabilities [47]. The W-ADL aims to measure the level of independence in performing typical daily activities including dressing, grooming, housework, meal-related activities, and activities outside the home [30].
- Barthel Index for the measurement of ADLs [48]. This index evaluates 10 activities: bathing, dressing, personal hygiene, use of the toilet, transfers (bed-armchair transfers), going up and down stairs, urinary and faecal continence, and feeding [32,36,42].
- Lawton IADL to measure the Instrumental Activities of Daily Living (IADL) [49]. This assesses eight instrumental activities: telephone use, money management, shopping, cooking, household chores, laundry, transportation use, and medication management [37,42,43].
- Functional Independence Measure (FIM) [50]. This assesses functional capacity in six areas—self-care, continence, mobility, transference, communication, and social cognition—with a total of 18 items [32,51].
- Resident Assessment Instrument-Home Care (RAI-HC) [52]. This is a standardised assessment tool to assess the health status of long-stay home care clients, the need for care, and basic information about housing and informal caregivers [53]. The RAI-HC includes elements related to demographic characteristics, home environment, functioning, health, medications, informal support, and formal health services [34,35].

The tools used to assess functional limitation refer to the scales that determine physical fitness, which include the assessment of gait, mobility and balance, mobility of the upper limbs, and physical activity.

Assessment of physical condition: gait, mobility, and balance.

- Hauser Ambulation Index (AI). This assesses the time and degree of assistance needed to walk 25 feet (independently, with a walker or wheelchair, or unable to move independently) [54].
- Gross Motor Function Classification Scale (GMFCS). This is a tool to measure walking ability (at home, at work/school, less than 50 m in a safe environment, more than 50 m in a safe environment, outside the safe environment) [54,55].
- Short Physical Performance Battery (SPPB). It is one of the most common measures of physical performance in the ageing population [56]. The SPPB consists of three subtests: balance (standing with feet together, in semi-tandem, and tandem positions), leg strength (rising from and sitting back down in an armless chair five times as quickly as possible), and walking speed over 4 m at a normal pace [26].
- 2-Minute Walk Test (2-MWT) [57]. It measures a person’s functional capacity and ability to walk at their own pace, particularly for those who are unable to take the longer 6-min walk test (6-MWT) or the 12-min walk test [32].
- Performance-Oriented Mobility Assessment (POMA I) [58,59]. This is used to assess balance and gait and identify adults at risk of falls [29,32].
- Berg Balance Scale (BBS). This contains 14 items, some of which are common to POMA I [29,36,37].
- Walking speed [60]. The time it takes a person to travel a specific distance (typically 4 m) at a comfortable or fast speed is recorded [29,36,37].
- 30-s Chair Stand Test (30 CST), for measuring muscle endurance [61]. It also assesses lower body strength and dynamic balance. It involves counting the number of times a person stands up and sits down in a chair within 30 s [29,36,37].
- Modified Back-Saver Sit and Reach (MBSSR), for measuring flexibility. This is an extended and modified version of the sit-and-reach test with back support [29,62]. It is executed unilaterally on a Swedish bench, where a 30-cm measuring ruler is placed, placing the unassessed leg on the ground with a hip flexion of approximately 90º [29,36,37].
- Incremental Shuttle Walk Test (ISWT), for measuring cardiorespiratory fitness [63]. The effort involves a physical condition that increases over time and consists of walking back and forth in a segment marked by two cones that are 9 m apart [29,36,37].

Assessment of physical condition: mobility of the upper limb.

- Box and Block Test (BBT) [64], for measuring gross manual dexterity. It consists of a box divided into two compartments, each containing 150 blocks. The task is to move as many blocks as possible from one compartment to the other within 60 s [29,36,37].
- Grip Strength (GS) [65], for measuring the grip force of the dominant hand, typically using dynamometry. For the seated patient, the dominant arm is assessed by flexing it to 90 degrees and holding the dynamometer while performing a maximum grip for three to five seconds, followed by a recovery time of 30 s between three attempts, taking into account the best result [29,36,37].

Assessment of physical fitness: physical activity.

- Pedometer [43] and accelerometer [26], for directly quantifying models of objective physical activity. These are motion sensors that record the number of steps and acceleration of the body, respectively.

### 3.2. Mental Assessment Tools

Among the cognitive assessment tools, it is important to distinguish between those used for screening, diagnosis, staging, and assessing behavioural or psychological symptoms.

#### Dementia Screening Tests

- Folstein Minimental State Examination (MMSE) [66]. This assesses orientation, memory, attention, concentration, calculation, language, and visuoconstructive skills [31,46].
- Montreal Cognitive Assessment (MoCA) [67]. This has been designed to assess mild cognitive dysfunctions. This instrument examines the following skills: attention, concentration, executive functions (including the ability to abstract), memory, language, visuoconstructive skills, calculation, and orientation [28].
- Wechsler Adult Intelligence Scale [WAIS-III] [68]; [WAIS-IV] [69]. This is a global intelligence scale that allows you to obtain the IQ (verbal, manipulative, and total) as well as four specific indices: verbal comprehension, perceptual organisation, working memory, and processing speed [27,31].
- Wechsler Memory Scale [WMS-III] [69,70]. The WMS-III is designed to assess the main aspects of memory functioning in adults aged between 16 and 89 years. It assesses episodic declarative memory—the ability to consciously store and retrieve specific aspects of information related to a particular situation or context—as well as working memory. The revised version of the WMS-III, the WMS-IV, incorporates the Brief Test for the Assessment of Cognitive Status (BCSE) as an additional test [45].
- Rey-Auditory Verbal Learning Test [RAVLT] [71]. This is a test developed by Rey (1964) that assesses immediate free recall, susceptibility to interference, short-term free recall, deferred free recall, and recognition [31,45].
- Controlled Oral Word Association [COWAT] [72]. This task involves the oral production of words based on phonetic instructions. Using the P, M, and R triad, the subject must recall all the words they know that begin with each of the letters in this triad within one minute [31,45].
- Groninger Intelligence Test 2 [GIT-2] [73]. This assesses semantic fluency. It involves naming as many words as possible within a specific category (e.g., animals, professions) in two 1-min attempts [31,45].
- Cognitive Failures Questionnaire [CFQ] [74]. This assesses the experience of memory errors, making errors, and distractibility in everyday situations [31,45,46].
- World Health Organization Disability Assessment Schedule 2.0 (WHODAS 2.0) [75]. This instrument measures the health and disability of adults over 18 years of age within a clinical or population context. It captures an individual’s level of functioning across six main life domains: comprehension and communication (cognition), movement (mobility), self-care (ability to maintain personal hygiene, dress, eat, and live independently), interpersonal interactions (social and interpersonal functioning), life activities (home, work, or school activities), and societal participation (engagement in family, social, and community activities) [39].
- Dementia Screening Questionnaire for Individuals with Intellectual Disabilities (DSQIID) [76]. This assesses ADLs and identifies social and cognitive impairments for early detection of dementia [8].
- Reiss Screen for Maladaptive Behaviour (RSMB) [77]. This scale assesses psychopathology in people with ID [8].
- Diagnostic Interview for Mental Disorders - Short Version (Mini-DIPS) [78]. The Mini-DIPS is the short form of this structured interview, designed according to DSM-IV and ICD-10 criteria, to assess current comorbidity (within 6 months) and covers the following disorders: anxiety, affective, somatisation, obsessive-compulsive, post-traumatic stress, acute stress, dissociative, and eating disorders [39].
- Delis Kaplan Executive Function System (D-KEFS) [79]. This psychometric test comprises nine independent assessments that comprehensively evaluate key components of executive functions, which are believed to be mainly mediated by the frontal lobe [27].
- The Zoo Map Test of the Behavioral Assessment of the Dysexecutive Syndrome (BADS Zoo Map) [80,81]. This is a battery of tests designed to assess the effects of dysexecutive syndrome, a set of deficiencies usually associated with damage to the frontal lobes of the brain [27].
- Wisconsin Card Sorting Task (WCST) [82]. This is used to assess abstraction capacity, concept formation, and the ability to adapt cognitive strategies in response to changes in environmental contingencies [27].
- Behavior Rating Inventory of Executive Function–Adult Version BRIEF-A [83]. This is a standardised measure that captures opinions about an adult’s executive functioning and daily self-regulation in their environment [27].
- Prospective and Retrospective Memory Questionnaire (PRMQ) [84]. This is a 16-item self-report questionnaire designed to assess prospective and retrospective memory problems in adults aged 18–93 years [41].

### 3.3. Biomedical/Clinical Assessment Tools

- Comorbid conditions. Sources include medical records, biological controls, and medical examinations. The physical examination covers cardiovascular, vascular, pulmonary, abdominal, neurological, ear, nose, and throat (ENT), skin, lymph nodes, thyroid, and assessment for orthostatic hypotension [8].
- Anthropometry. This covers height, weight, and body mass index (BMI; kg m^−2^) [26].
- Pharmacological treatment. This involves the total number of medicines used [8,38].
- Hospitalisation occurrences. Hospitalisation is defined as a stay of at least one day in a standard hospital [38].
- Drug Burden Index (DBI) [85]. The DBI is defined as the sum of the anticholinergic and sedative effects for each prescribed medication and is indirectly related to the use of psychotropic drugs [8].
- Assessment of the pace of ageing. This includes ageing biomarkers [86,87], facial ageing, and perceived health. Nineteen biomarkers cover the main aspects of ageing: body mass index, waist-to-hip ratio, glycosylated haemoglobin, leptin, mean arterial pressure, cardiorespiratory fitness, forced expiratory volume in 1 s (FEV1), FEV1/forced vital capacity ratio, total cholesterol, triglycerides, high-density lipoprotein cholesterol, apolipoprotein B100/A1 ratio, lipoprotein (a), creatinine clearance, urea nitrogen, C-reactive protein, white blood cell count, average periodontal attachment loss, and affected tooth decay or surfaces. Perceived health is assessed through self-reports, informants’ impressions, and interviewer’s impressions [7].

### 3.4. Social Valuation Tools

- Vineland Adaptive Behavior Scales, Second Edition (VABS-II) [88]. This evaluates a multitude of aspects across five domains: communication, daily living skills, socialisation, motor skills, and adaptive behaviour index [8].

### 3.5. Other Domains

#### 3.5.1. Fragility

- Intellectual Disability-Frailty Index (ID-FI) and ID-FI Short Form. In the Healthy Ageing and Intellectual Disability (HA-ID) study, baseline data were collected across three subtopics: physical activity and fitness, nutrition and nutritional status, and mood and anxiety. A practical tool was developed to assess frailty in individuals with ID [38].

#### 3.5.2. Risk of Falls

- The Johns Hopkins Fall Risk Assessment Tool (JHFRAT) [89]. This composite scale comprises eight areas of assessment, classifying each risk factor for falls as follows: previous defining situations of risk, which include immobilisation (low risk), history of falls (high risk), history of falls during hospitalisation (high risk), and whether the patient is classified as high risk according to the protocols (high risk); age; medication; healthcare equipment; mobility; and cognition [26].

#### 3.5.3. Quality of Life

- World Health Organization Quality-of-Life Scale (WHOQOL-BREF) [90]. This is an abbreviated version of the original WHOQOL tool. It contains 26 items: two related to overall quality of life and satisfaction with health, and 24 grouped into four areas: physical health, psychological health, social relations, and environment [25].
- Quality of Life Questionnaire (QoL-Q) [91]. This measures the quality of life of people with ID. The following dimensions are assessed: satisfaction with personal life, competence and productivity, empowerment and independence, and social belonging and community integration [25].
- Quality of Life Inventory (QOLI) [92]. This 32-item tool measures quality of life in four domains: health, relationships, employment, and living conditions [25].
- Comprehensive Quality of Life Inventory (ComQOL) [93]. This 35-item tool assesses quality of life across seven domains: material well-being, health, productivity, privacy, security, place in the community, and emotional well-being [25].
- Short Form Health Survey (SF-36) [94]. This 36-item tool assesses quality of life in eight domains: physical health, physical role, body pain, overall health, vitality, social function, emotional role, and mental health [8,25].
- Medical Outcomes Study Short Form Health Survey Version 2 (SF-12 v.2) [95]. This is a shortened 12-item version of the SF-36, where items are grouped into the summary of the physical component and the summary of the mental component [25].
- Novel QoL measures (QOL1 and QOL2) [96]. This is an indirect assessment of the ‘autism-friendly environment’ and includes 5 items: staff/caregivers’ knowledge of autism, the application of structured education, the implementation of an individual treatment/training plan, the degree to which daily living/employment is appropriate to an individual’s ability, and the overall level of quality of life [25].
- Assessment of life satisfaction ‘Fragebogen zur Lebenszufriedenheit’ (FLZ) [97]. This assesses life satisfaction in various areas, including health; work and employment; financial situation; leisure; relationships with partners, one’s own children, friends, and family; sexuality; and housing [39].
- Autism-Specific Quality of Life Questionnaire (ASQoL) [33]. This is a set of nine items developed to specifically assess the quality of life in autistic individuals. It serves to complement general quality of life instruments, such as the WHOQoL-BREF, by capturing aspects of well-being that are particularly relevant to the autistic population [41].

## 4. Discussion

Fifty-seven tools have been identified for performing a CGA, of which 34 have been used with people with ASD, particularly focusing on assessing the functional [30], mental [8,27,28,30,31,39,41,45,46], biomedical [7,8], social [8], and quality of life domains [25,30,41]. A greater number of tools correspond to the mental domain and quality of life, while only one tool has been identified in both the functional domain (W-ADL) and the social domain (VABS-II).

The 23 tools used for people with ID focused on assessing functional domains [26,29,32,35,36,37,42,43], biomedical factors [26,38], fragility [33,34,38,40,42,43], quality of life [26], and the risk of falls [26], highlighting the predominance of tools in the functional domain. In contrast, no tools have been identified to assess the mental and social domains.

Regarding the mental domain, various authors [8,27,28,30,31,39,41,45,46] have used numerous tools that assess the cognitive functions of people with ASD. However, no tools have been observed that assess affective states. 

Concerning the assessment of the social domain, the only tool specifically focused on this area is the VABS-II [8]. However, social relationships are also considered in quality of life assessments [25,39], including QOL1 and QOL2, which were validated by Billstedt et al. [96] in people with ASD.

While most of the identified scales are limited to assessing a single domain within the geriatric assessment, three tools (FIM, RAI-HC, and WHODAS 2.0) perform a generic assessment that includes factors from different domains. Schmidt et al. [39] used the WHODAS tool to assess aspects such as self-care, mobility, activities of daily living, and cognitive function in people with ASD. Likewise, the ASQoL [33] addresses issues, such as sensory overload, lack of financial security, and barriers healthcare access, that are particularly relevant for autistic individuals. A combination of this tool with others such as the WHODAS could constitute an approach to a comprehensive geriatric assessment for autistic people. The other two tools, FIM [32] and RAI-HC [34,35], were used with individuals with ID.

Other tools used by Schoufour, Echteld, et al. [38]; Schoufour, Evenhuis, et al. [42]; Schoufour, Mitnitski, et al. [43]; and Choi et al. [26] also perform a more generic assessment of people with ID. Specifically, the ID-FI and ID-FI Short Form (frailty) and the JHFRAT (risk of falls) assess factors from different domains of the CGA.

In this review, we did not find any tool that focuses on assessing all domains necessary for a complete CGA in people with ASD. However, the ‘multi-domain’ tools used by Schoufour, Echteld, et al. [38]; Schoufour, Evenhuis, et al. [42]; Schoufour, Mitnitski, et al. [43]; and Choi et al. [26] to assess individuals with ID could serve as a reference point for conducting a more complete and practical geriatric assessment. 

### Limitations and Strengths

Among the limitations, the studies included in this review did not uniformly define the geriatric age range for people with ASD. Furthermore, since ID may or may not be present in people with ASD, this term was included in the search strategy to identify all tools that could be useful for the comprehensive assessment of people with ASD throughout the ageing process.

As a strength, this study emphasises the importance of considering the individual as a whole, aiming to provide a broad perspective of the tools available for addressing ageing in autism.

## 5. Conclusions

This review identified a total of 57 tools that are used to assess the various domains of ageing in individuals with ASD, as well as in those with ID, within the framework of the CGA. Most of these tools focus on specific domains, particularly the mental and quality-of-life domains in individuals with ASD and the functional domain in individuals with ID.

However, no tool specifically designed for individuals with ASD was found that comprehensively addresses all domains relevant to a CGA. Some multi-domain instruments, such as the WHODAS 2.0, FIM, and RAI-HC, have demonstrated the ability to assess general the aspects of functioning and could serve as a foundation for developing more comprehensive instruments. Similarly, scales such as the ID-FI or the JHFRAT, used in individuals with ID, represent promising approaches for capturing the various dimensions of ageing.

The combination of these tools with ASD-specific instruments such as ASQoL—which addresses the characteristics unique to autistic individuals—may represent an initial step towards the development of a tool that is truly tailored to the needs of this population.

Therefore, it is necessary to develop a multi-domain tool specifically for individuals with ASD that includes evaluations of the main geriatric domains (functional, mental, clinical, social, frailty, fall risk, and quality of life) as well as the autism-specific aspects of ageing. This more holistic and practical approach will not only benefit clinical practice, but also guide future research, facilitate the standardisation of geriatric assessment in this population, and promote more personalised and effective care.

## Figures and Tables

**Table 1:** Inclusion and exclusion criteria.

INCLUSION
Studies including adults aged 40 years or older with ASD or ID
EXCLUSION
Pathology: Down syndrome, Alzheimer’s and other dementias, Hunter’s disease, cancer, Rett syndrome, COVID-19
Life stage: childhood stage
Type of study: experimental with animals, study protocol, case study, intervention studies
Topic of study: brain structures, palliative care in people with intellectual disabilities, caregivers, diagnostic tools and characteristics of ASD, assessment of isolated cognitive function, socio-health resources

**Table 2:** Characteristics of the selected studies.

Author (Year)/Country	Study Design	Assessment	Participants and Range Years or Mean Age	Key Findings
Ayres et al. (2018)/UK [25]	Systematic review	WHOQOL-BREF QoL-Q QOLI ComQOL SF-36 SF-12 v.2 QOL1 and QOL2	N = 959 18–83 years	No comprehensive, autism spectrum disorder–specific quality of life measurement tools have been validated.
Choi et al. (2020)/Southeast of the United States [26]	Cohort study	Anthropometrics JHFRAT SF-36 SPPB Accelerometer	N = 80 43 ± 13 years	Adults with ID who experience falls are more likely to need support with ADLs, be older, and have arthritis, rheumatism, and walking problems than adults with ID who do not experience falls.
Geurts et al (2020)/Netherlands [27]	Cross-sectional	WAIS IV D-KEFS BADS Zoo Map WCST Adult BRIEF-A	N = 101 60–85 years	Subjective measures offer valuable insight into everyday executive functioning and the experienced problems in an ASC population.
Groot et al. (2021)/Netherlands [28]	Case-control	MMSE-NL MoCA-NL	N = 100 30–73 years	There is no difference in performance between people with and without an ASC on the MMSE-NL or MoCA-NL.
Hilgenkamp et al. (2010)/Netherlands [29]	Systematic review	BBT BBS POMA I Walking speed GS 30 CST MBSSR ISWT	Older adults (age is not specified)	The following are proposed for measuring physical fitness: BBT, reaction time test, BBS, walking speed, GS, 30 CST, MBSSR, and ISWT.
Hwang et al. (2020)/Australia [30]	Cross-sectional	W-ADL SF-12 WHODAS 2.0	N = 152 40–79 years	Significantly less autistic adults were ‘maintaining physical and cognitive functioning’ and ‘actively engaging with life’ in comparison to controls. The current dominant model of ‘ageing well’ is limited for examining autistic individuals.
Lever and Geurts (2016)/Netherlands [31]	Cross-sectional	WAIS III MMSE WMS-III RAVLT COWAT GIT-2 CFQ	N = 236 20–79 years	Age-related differences characteristic of typical ageing are reduced or parallel, but not increased, in individuals with ASD.
Maring et al. (2013)/USA [32]	Systematic review and pilot study	Barthel Index FIM POMA I 2-MWT	N = 30 >50 years	The measures are strongly associated and successfully distinguished between participants with an adverse health event in the previous year.
Mason et al. (2021)/New Zealand [7]	Cohort design	Pace of ageing: ageing biomarkers, facial ageing, perceived health	N = 915 3–45 years	They found that higher autistic traits were associated with poorer physical health and a faster pace of ageing.
McConachie et al. (2018)/UK [33]	Validation study	ASQoLç WHOQoL-BREF	N = 309 18–76 years	A psychometric validation of the World Health Organization measure WHOQoL-BREF was conducted; additionally, the construct validity of the WHO Disabilities module was examined, and nine additional autism-specific items (ASQoL) were developed based on extensive consultation with the autism community.
McKenzie (2016)/Ontario Canada [34]	Cohort design	RAI-HC	N = 3034 18–99 years	Frail individuals had greater rates of admission than non-frail individuals. The FI predicts institutionalisation.
McKenzie et al. (2015)/Ontario, Canada [35]	Cohort study	RAI-HC	N = 7863 18–99 years	Using the FI to identify frailty in adults with IDD is feasible and may be incorporated into existing home care assessments.
Miot et al. (2023)/France [8]	Cohort design	VABS-II Total number of medications DBI Comorbidities DSQIID RSMB	N = 63 25–59 years	Spectrum disorder + intellectual disability individuals can be identified based on their multimorbidity and potentially different ageing trajectories.
Oppewal et al. (2014)/Netherlands [36]	Cohort design	BBT BBS Walking speed GS 30 CST MBSSR ISWT	N = 602 >50 years	Physical fitness significantly predicts a decline in daily functioning in older adults with ID.
Oppewal et al. (2015)/Netherlands [37]	Cohort design	BBT BBS Walking speed GS 30 CST MBSSR ISWT Lawton IADL	N = 601 >50 years	Physical fitness is found to be an important aspect for IADL.
Roestorf et al. (2025)/UK [38]	Cross-sectional	PRMQ EBPM TBPM WHOQOL-BREF	N = 57 23–80 years	QoL was positively associated with TBPM accuracy in non-autistic participants. In addition to confirming previous findings showing that autistic individuals have more significant difficulties with TBPM compared to EBPM, the results suggest that neither difficulties with EBPM nor TBPM appear to adversely affect their overall or health-related QoL.
Schmidt et al. (2015)/Germany [39]	Cross-sectional	Mini-DIPS WHODAS 2.0 FLZ	N = 87 Age: mean = 31	Adults on the autism spectrum without intellectual impairment experience significant functional impairments in social domains, but they are relatively competent in daily living skills.
Schoufour et al. (2022)/Netherlands [40]	Longitudinal and case series	ID-FI ID-FI Short Form	N = 982 >50 years	A practical tool to assess the frailty status of people with ID is introduced.
Schoufour, Echteld, et al. (2015)/Netherlands [41]	Cohort design	Occurrences of hospitalisation Total number of used medicines Comorbid conditions ID-FI	N = 982 >50 years	The FI was related to an increased risk of higher medication use and several comorbid conditions, although not to falls, fractures, and hospitalisation.
Schoufour, Evenhuis, et al. (2014)/Netherlands [42]	Cohort design	Barthel Index Lawton IADL ID-FI	N = 676 >50 years	Increased care during the follow-up was related to a high frailty index score at baseline.
Schoufour, Mitnitski, et al. (2014)/Netherlands [43]	Cohort design	Barthel Index Lawton IADL AI GMFCS ID-FI Pedometer	N = 703 >50 years	The FI demonstrated the highest predictive value for individuals with high baseline mobility or independence in IADLs.
Schoufour, Mitnitski, et al. (2015)/Netherlands [44]	Cohort design	-ID-FI	N = 982 >50 years	The predictive validity of the FI was strongly associated with 3-year mortality.
Torenvliet et al. (2022)/Netherlands [45]	Cohort design	RAVLT WMS-III COWAT GIT-2 CFQ	N = 176 30–89 years	Previously observed difficulties in Theory of Mind and verbal fluency, which appear to persist into older age, were replicated.
Torenvliet et al. (2023)/Netherlands [46]	Cohort design	CFQ WAIS III/IV MMSE	N = 464 24–85 years	Autistic individuals diagnosed in adulthood, without intellectual disability, do not seem at risk for accelerated cognitive decline.

2-MWT: 2-Minute Walk Test; 30 CST: 30-s Chair Stand Test; ADL: Activities of Daily Living; AI: Hauser Ambulation Index; ASC: Autism Spectrum Condition; ASQoL: Autism-Specific Quality of Life Questionnaire; BADS Zoo Map: The Zoo Map Test of the Behavioural Assessment of the Dysexecutive Syndrome; BBS: Berg Balance Scale; BBT: Box and Block Test; BRIEF-A: Behaviour Rating Inventory of Executive Function–Adult Version; CFQ: Cognitive Failures Questionnaire; ComQOL: Comprehensive Quality of Life Inventory; COWAT: Controlled Oral Word Association; DBI: Drug Burden Index; D-KEFS: Delis–Kaplan Executive Function System; DSQIID: Dementia Screening Questionnaire for Individuals with Intellectual Disabilities; FI: Frailty Index; FIM: Functional Independence Measure; FLZ: Assessment of life satisfaction ‘Fragebogen zur Lebenszufriedenheit’; GIT-2: Groninger Intelligence Test 2; GMFCS: Gross Motor Function Classification Scale; GS: Grip Strength; IADL: Instrumental Activities of Daily Living; IDD: Intellectual and Developmental Disabilities; ID-FI: Intellectual Disability-Frailty Index; ISWT: Incremental Shuttle Walk Test; JHFRAT: The Johns Hopkins Fall Risk Assessment Tool; Lawton IADL: Lawton Instrumental Activities of Daily Living; MBSSR: Modified Back-Saver Sit and Reach; Mini-DIPS: Diagnostic Interview for Mental Disorders—Short Version; MMSE: Folstein Minimental State Examination; MoCA: Montreal Cognitive Assessment; POMA I: Performance-Oriented Mobility Assessment; PRMQ: Prospective and Retrospective Memory Questionnaire; QoL: Quality of Life; QOL1 and QOL2: Novel QoL measures; QOLI: Quality of Life Inventory; QoL-Q: Quality of Life Questionnaire; RAI-HC: Resident Assessment Instrument-Home Care; RAVLT: Rey-Auditory Verbal Learning Test; RSMB: Reiss Screen for Maladaptive Behaviour; SF-12 v.2: Medical Outcomes Study Short-Form Health Survey Version 2; SF-36: Short-Form Health Survey; SPPB: Short Physical Performance Battery; VABS-II: Vineland Adaptive Behaviour Scales, Second Edition; W-ADL: Waisman Activities of Daily Living Scale; WAIS-III, WAIS-IV: Wechsler Adult Intelligence Scale; WCST: Wisconsin Card-Sorting Task; WHO: World Health Organization; WHODAS 2.0: World Health Organization Disability Assessment Schedule 2.0; WHOQOL-BREF: World Health Organization Quality-of-Life Scale; WMS-III: Wechsler Memory Scale.

## Data Availability

No new data were created or analyzed in this study. Data sharing is not applicable to this article.

## Supplementary Materials

The following supporting information can be downloaded at: https://www.mdpi.com/article/10.3390/healthcare13202640/s1, Supplementary File S1: Search Strategy; Supplementary File S2: List of the Excluded Studies at Full-Text Assessment; Supplementary File S3: JBI’s Critical Appraisal Tools; Supplementary File S4: Key Assessment Tools by Domain: Availability and Psychometric Properties.
