# Supplementary material for: Tools for Detecting Ageing in People with Autism Spectrum Disorder: A Scoping Review
Source: Healthcare (Basel). 2025 Oct 20;13(20):2640. doi: 10.3390/healthcare13202640 (PMC12563972; doi:10.3390/healthcare13202640)
Supplement: Supplementary file 1 [file healthcare-13-02640-s001.zip › healthcare-3787181-supplementary.pdf]

Supplementary File S1. Search Strategy

Database	Terms	Filters	Results
Cochrane	Advanced search with the term “autism” in title, abstract, keyword	-	27
NICE	Search with the term “autism”	-	91
PsicInfo	((Autism Spectrum Disorder) AND (Intellectual Disability)) AND ((Aging) OR (Frailty) OR (Frail Elderly)) AND (assessment)	Data: 2003–2023	16
Pubmed	(((“Autism Spectrum Disorder”[Mesh]) OR (“Intellectual Disability”[Mesh])) AND (((“Aging”[Mesh]) OR (“Frailty”[Mesh])) OR (“Frail Elderly”[Mesh]))) AND (assessment)	Data: 2003–2023	172
Scopus	(KEY(aging) OR KEY(frailty) OR KEY(frail AND elderly) AND KEY(assessment) AND KEY(autism) OR KEY(intellectual AND disability))	Data: 2003–2023	238
WOS	Topic (((ALL = (Autism Spectrum Disorder)) OR ALL = (intellectual disability)) AND (ALL = (aging OR frailty OR frail elderly)) AND ALL = (assessment)	Data: 2003–2023<br>Web of Science Categories: Medicine General Internal or Geriatrics Gerontology or Gerontology or Nursing or Primary Health Care or Health Care Sciences Services or Psychology Clinical	163

Supplementary File S2. List of the Excluded Studies at Full-Text Assessment

	Study	Title	Reasons for Exclusion
1	Abbott et al. (2018)	Exploratory Study of Executive Function Abilities Across the Adult Lifespan in Individuals Receiving an ASD Diagnosis in Adulthood	Evaluation of a partial function
2	Alcedo et al. (2017)	People with intellectual disability who are ageing: Perceived needs assessment	Focused on health resources and quality of life

3	Amanullah et al. (2019)	An overview of autism in the elderly	Diagnostic Tools for Autism Spectrum Disorder (ASD)
4	Baron-Cohen et al. (2001)	The Autism-Spectrum Quotient (AQ): Evidence from Asperger syndrome/high-functioning autism, males and females, scientists and mathematicians	Focused on ASD characteristics
5	Beck et al. (2020)	Assessment and Treatment of Emotion Regulation Impairment in Autism Spectrum Disorder Across the Life Span: Current State of the Science and Future Directions	Focused on ASD characteristics
6	Chitty et al. (2016)	Central nervous system medication use in older adults with intellectual disability: Results from the successful ageing in intellectual disability study	Focused on health resources and quality of life
7	De Vreese et al. (2021)	Short forms of Prudhoe Cognitive Function Test in adults and aging people with intellectual disabilities: Italian validation study	Focused on other pathologies or syndromes
8	Edelson et al. (2021)	Strategies for Research, Practice, and Policy for Autism in Later Life: A Report from a Think Tank on Aging and Autism.	Focused on ASD characteristics
9	Howlin and Magiati (2017)	Autism spectrum disorder outcomes in adulthood	Focused on ASD characteristics
10	Huang et al. (2023)	Experiences of performing daily activities in middle aged and older autism adults: a qualitative study	Focused on health resources and quality of life
11	Jahan and Ismayilova (2018)	Important considerations in the assessment of seniors who are aging with cognitive or intellectual disabilities	Diagnostic Tools for ASD
12	Kovačič et al. (2020)	The impact of multicomponent programmes on balance and fall reduction in adults with intellectual disabilities: a randomised trial	Intervention study
13	Kuschner and Malow (2021)	Autism and physical health across the lifespan	Focused on health resources and quality of life
14	Lehmann et al. (2013)	Ageing with an intellectual disability: the impact of personal resources on well-being	Focused on health resources and quality of life
15	Lever and Geurts (2018)	Is Older Age Associated with Higher Self- and Other-Rated ASD Characteristics?	Diagnostic Tools for ASD

16	Martínez Aldao et al. (2020)	Effects of a physical exercise programme on the risk of falls, balance, and walking speed in older people with intellectual disabilities	Intervention study
17	Mason et al. (2019)	A Systematic Review of What Barriers and Facilitators Prevent and Enable Physical Healthcare Services Access for Autistic Adults	Focused on health resources and quality of life
18	Merrick et al. (2006)	Aging with autism	Focused on ASD characteristics
19	Mukaetova-Ladinska et al. (2011)	Ageing in people with autistic spectrum disorder	Focused on other pathologies or syndromes
20	National Institute for Health and Care excellence [NICE] (2014)	Autism (NICE guidelines QS51)	Focused on ASD characteristics
21	National Institute for Health and Care excellence [NICE] (2021)	Surveillance of autism (NICE guidelines CG128, CG142 and CG170) [Internet]. London: National Institute for Health and Care Excellence (NICE); 14 June 2021. Available from: https://www.ncbi.nlm.nih.gov/books/NBK571333/ (accessed on 8 January 2024).	Focused on ASD characteristics
22	Oppewal et al. (2013)	Feasibility and outcomes of the Berg Balance Scale in older adults with intellectual disabilities	Evaluation of a partial function
23	Ouellette-Kuntz et al. (2019)	How best to support individuals with IDD as they become frail: Development of a consensus statement	Evaluation of a partial function
24	Racine et al. (2022)	Challenges in assessing change in autistic adults: scale limitations and discrepancies in reporting in clinical trials	Diagnostic Tools for ASD
25	Roestorf et al. (2019)	"Older Adults with ASD: The Consequences of Aging." Insights from a series of special interest group meetings held at the International Society for Autism Research 2016–2017	Focused on health resources and quality of life
26	Ruggieri (2022)	El autismo a lo largo de la vida	Focused on ASD characteristics

27	Ruggieri et al. (2019)	Aging and autism: Understanding, intervention and proposals to improve quality of life	Focused on health resources and quality of life
28	Rydzewska et al. (2018)	Prevalence of long-term health conditions in adults with autism: observational study of a whole country population	Focused on health resources and quality of life
29	Schepens et al. (2018)	How to improve the quality of life of elderly people with intellectual disability: A systematic literature review of support strategies	Focused on health resources and quality of life
30	Service and Hahn. (2003)	Issues in aging The role of the nurse in the care of older people with intellectual and developmental disabilities	Focused on health resources and quality of life
31	Steward et al. (2020)	The Mental and Physical Health Profiles of Older Adults Who Endorse Elevated Autistic Traits	Focused on ASD characteristics
32	Tena-Bernal et al. (2021)	Cognitive and Functional Differences in Aging with and without Intellectual Disabilities: Observational Study	Evaluation of a partial function
33	Thalen et al. (2020)	Psychosocial interventions for older people with intellectual disabilities and the role of support staff: A systematic review	Intervention study
34	Thom et al. (2020)	Psychiatric assessment of social impairment across the lifespan.	Diagnostic Tools for ASD
35	Torres-Unda et al. (2017)	The Feldenkrais Method improves functioning and body balance in people with intellectual disability in supported employment: A randomized clinical trial	Intervention study
36	Tse et al. (2018)	Ageing in individuals with intellectual disability: issues and concerns in Hong Kong	Focused on other pathologies or syndromes
37	Wallace et al. (2016)	Aging and autism spectrum disorder: Evidence from the broad autism phenotype	Focused on ASD characteristics
38	Wise (2020)	Aging in Autism Spectrum Disorder	Focused on ASD characteristics

Supplementary File S3. JBI's Critical Appraisal Tools

A. Assessment for bias using JBI critical appraisal checklist for cohort studies

Questions

1. Were the two groups similar and recruited from the same population?
2. Were the exposures measured similarly to assign people to both exposed and unexposed groups?
3. Was the exposure measured in a valid and reliable way?
4. Were confounding factors identified?
5. Were strategies to deal with confounding factors stated?
6. Were the groups/participants free of the outcome at the start of the study (or at the moment of exposure)?
7. Were the outcomes measured in a valid and reliable way?
8. Was the follow up time reported and sufficient to be long enough for outcomes to occur?
9. Was follow up complete, and if not, were the reasons to loss to follow up described and explored?
10. Were strategies to address incomplete follow up utilized?
11. Was appropriate statistical analysis used?

**Cohort Study**	**Questions**											
	**1**	**2**	**3**	**4**	**5**	**6**	**7**	**8**	**9**	**10**	**11**	**JBI Score**
Choi et al. (2020) [43]	+	+	+	+	+	+	+	Not applicable	+	+	+	10
Mason et al. (2021) [7]	Not applicable	Not applicable	+	+	Unclear	+	+	+	-	-	+	6
McKenzie et al. (2015) [38]	Not applicable	Not applicable	+	+	+	+	+	+	+	+	+	9
McKenzie et al. (2016) [39]	Not applicable	Not applicable	+	+	Unclear	+	+	+	+	+	+	8
Miot et al. (2023) [8]	+	+	+	+	+	+	+	+	+	+	+	11

Oppewal et al. (2014) [29]	Not applicable	Not applicable	+	+	+	+	+	+	+	+	+	9
Oppewal et al. (2015) [32]	Not applicable	Not applicable	+	+	+	+	+	+	+	+	+	9
Schoufour, Echteld et al. (2015) [79]	Not applicable	Not applicable	+	+	+	+	+	+	+	+	+	9
Schoufour, Evenhuis et al. (2014) [30]	Not applicable	Not applicable	+	+	+	+	+	+	+	+	+	9
Schoufour, Mitnitski et al. (2014) [33]	Not applicable	Not applicable	+	+	+	+	+	+	+	+	+	9
Schoufour, Mitnitski et al. (2015) [96]	Not applicable	Not applicable	+	+	+	+	+	+	+	+	+	9
Torenvliet et al. (2022) [63]	+	+	+	+	+	+	+	+	+	Not applicable	+	10
Torenvliet et al. (2023) [56]	+	+	+	+	+	+	+	+	+	+	+	11

"+" = Yes, the study meets the criterion; "–" = No, the study does not meet the criterion.

Aromataris, E.; Munn, Z. (Eds.). JBI Manual for Evidence Synthesis. JBI, 2020. Available from https://synthesismanual.jbi.global (accessed on 25 January 2024). https://doi.org/10.46658/JBIMES-20-01.

B. Assessment for bias using JBI critical appraisal checklist for systematic reviews

Questions

1. Is the review question clearly and explicitly stated?
2. Were the inclusion criteria appropriate for the review question?

3. Was the search strategy appropriate?
4. Were the sources and resources used to search for studies adequate?
5. Were the criteria for appraising studies appropriate?
6. Was critical appraisal conducted by two or more reviewers independently?
7. Were there methods to minimize errors in data extraction?
8. Were the methods used to combine studies appropriate?
9. Was the likelihood of publication bias assessed?
10. Were recommendations for policy and/or practice supported by the reported data?
11. Were the specific directives for new research appropriate?

**Systematic Reviews**	**Questions**											
	**1**	**2**	**3**	**4**	**5**	**6**	**7**	**8**	**9**	**10**	**11**	**JBI Score**
Ayres et al. (2018) [87]	Unclear	+	+	+	+	+	+	+	-	+	+	9
Hilgenkamp et al. (2010) [47]	Unclear	+	+	+	+	Unclear	-	+	-	+	+	7
Maring et al. (2013) [28]	Unclear	+	+	+	+	+	-	+	-	+	+	8

"+" = Yes, the study meets the criterion; "–" = No, the study does not meet the criterion.

Aromataris E, Fernandez R, Godfrey C, Holly C, Kahlil H, Tungpunkom P. Summarizing systematic reviews: methodological development, conduct and reporting of an Umbrella review approach. Int J Evid Based Healthc. 2015;13(3):132–40. Available from https://synthesismanual.jbi.global (accessed on 25 January 2024). https://doi.org/10.46658/JBIMES-20-11.

C. Assessment for bias using JBI critical appraisal checklist for analytical cross-sectional studies

Questions

1. Were the criteria for inclusion in the sample clearly defined?

2. Were the study subjects and the setting described in detail?
3. Was the exposure measured in a valid and reliable way?
4. Were objective, standard criteria used for measurement of the condition?
5. Were confounding factors identified?
6. Were strategies to deal with confounding factors stated?
7. Were the outcomes measured in a valid and reliable way?
8. Was appropriate statistical analysis used?

**Analytical Cross-sectional Studies**	**Questions**								
	**1**	**2**	**3**	**4**	**5**	**6**	**7**	**8**	**JBI Score**
Geurts et al. (2020) [61]	+	+	+	+	+	+	+	+	8
Hwang et al. (2020) [26]	+	+	+	+	+	+	+	+	8
Lever and Geurts (2016) [55]	+	+	+	+	+	+	+	+	8
Schmidt et al. (2015) [69]	Unclear	+	+	+	+	+	+	+	7

"+" = Yes, the study meets the criterion; "–" = No, the study does not meet the criterion.

Aromataris E, Munn Z (Editors). JBI Manual for Evidence Synthesis. JBI, 2020. Available from https://synthesismanual.jbi.global (accessed on 25 January 2024). https://doi.org/10.46658/JBIMES-20-01.

D. Assessment for bias using JBI critical appraisal checklist for analytical case–control studies

Questions

1. Were the groups comparable other than the presence of disease in cases or the absence of disease in controls?
2. Were cases and controls matched appropriately?
3. Were the same criteria used for identification of cases and controls?

4. Was exposure measured in a standard, valid and reliable way?
5. Was exposure measured in the same way for cases and controls?
6. Were confounding factors identified?
7. Were strategies to deal with confounding factors stated?
8. Were outcomes assessed in a standard, valid and reliable way for cases and controls?
9. Was the exposure period of interest long enough to be meaningful?
10. Was appropriate statistical analysis used?

**Case–Control Studies**	**Questions**										
	**1**	**2**	**3**	**4**	**5**	**6**	**7**	**8**	**9**	**10**	**JBI Score**
Groot et al. (2020) [58]	+	+	+	+	+	+	+	+	+	+	10

"+" = Yes, the study meets the criterion; "–" = No, the study does not meet the criterion.

Aromataris E, Munn Z (Editors). JBI Manual for Evidence Synthesis. JBI, 2020. Available from https://synthesismanual.jbi.global (accessed on 25 January 2024). https://doi.org/10.46658/JBIMES-20-01.

E. Assessment for bias using JBI critical appraisal checklist for analytical case series studies

Questions

1. Were there clear criteria for inclusion in the case series?
2. Was the condition measured in a standard, reliable way for all participants included in the case series?
3. Were valid methods used for identification of the condition for all participants included in the case series?
4. Did the case series have consecutive inclusion of participants?
5. Did the case series have complete inclusion of participants?
6. Was there clear reporting of the demographics of the participants in the study?
7. Was there clear reporting of clinical information of the participants?
8. Were the outcomes or follow up results of cases clearly reported?
9. Was there clear reporting of the presenting site(s)/clinic(s) demographic information?
10. Was statistical analysis appropriate?

Case Series Studies	Questions										
	1	2	3	4	5	6	7	8	9	10	JBI Score
Schoufour et al. (2022) [97]	+	+	+	-	+	+	+	+	+	+	9

"+" = Yes, the study meets the criterion; "–" = No, the study does not meet the criterion.

Munn Z, Barker TH, Moola S, Tufanaru C, Stern C, McArthur A, Stephenson M, Aromataris E. Methodological quality of case series studies: an introduction to the JBI critical appraisal tool. JBI Evidence Synthesis. 2020;18(10):2127–2133.

Supplementary File S4. Key Assessment Tools by Domain: Availability and Psychometric Properties

Domain	Tool	Availability	Main Psychometric Properties
**Functional Assessment**	W-ADL	Free	Acceptable reliability; content validity in disability
	Barthel Index	Free	High reliability (α 0.70–0.94); ICC 0.77–0.98; convergent validity; responsive to change
	Lawton IADL	Free	Construct validity; test–retest reliability; discriminative in older adults
	FIM	License required	Inter-rater reliability >0.90; validity and sensitivity in rehabilitation
	RAI-HC	Free (Software requires license)	Construct validity; useful in care planning
	Hauser AI	Free	Acceptable reliability; validity in multiple sclerosis

	GMFCS	Free	High inter-rater reliability; validity in cerebral palsy
	SPPB	Free	Reliability >0.80; predictive validity for frailty
	2/6/12-MWT	Free	Reliability >0.90; predictive validity for function and mortality
	POMA (Tinetti)	Free	Moderate–high reliability; validity for fall risk
	Berg Balance Scale	Free	ICC reliability >0.95; convergent validity in balance
	Walking Speed (4m)	Free	ICC reliability >0.90; valid predictor of disability
	30-s Chair Stand Test	Free	Reliability >0.80; validity for lower limb strength
	MBSSR	Free	Acceptable reliability; validity in functional flexibility
	ISWT	Free	Reliability >0.80; validity in cardiorespiratory capacity
	BBT	Free	ICC >0.90; validity in manual dexterity
	Grip Strength	Free	Excellent reliability; valid predictor of frailty
	Pedometer/Accelerometer	Free/Commercial	High objective validity; reliability depends on device
**Mental Assessment**	MMSE	License required	Acceptable reliability; established validity in dementia
	MoCA	Free with permission	High sensitivity in mild impairment; adequate reliability
	WAIS-III/IV	Commercial/license	Reliability >0.90; strong construct validity
	WMS-III	Commercial/license	Acceptable reliability; validity in episodic and working memory
	RAVLT	Free	Acceptable reliability; validity in verbal memory
	COWAT	Free	High reliability; validity in executive functions

	GIT-2	Commercial	Good reliability; validity in general intelligence
	CFQ	Free	Moderate reliability; validity as a self-report measure
	WHODAS 2.0	Free	Reliability α >0.80; cross-cultural validity
	DSQIID	Free	Validity in early dementia detection
	RSMB	Free with permission	Acceptable reliability; clinical validity in ID
	Mini-DIPS	Commercial/license	High reliability; diagnostic validity
	D-KEFS	Commercial	Adequate reliability; validity in executive functions
	BADS Zoo Map	Commercial	Acceptable reliability; validity in dysexecutive syndrome
	WCST	Free	Moderate reliability; demonstrated validity in cognitive flexibility
	BRIEF-A	Commercial	Reliability α >0.80; ecological validity
	PRMQ	Free	Reliability α >0.80; validity in prospective and retrospective memory in adults and clinical populations
**Social Assessment**	Vineland-II	Commercial	Excellent reliability; validity in adaptive behavior
**Other Domains**	ID-FI	Free	Predictive validity of frailty in ID
	JHFRAT	Free	Acceptable reliability; predictive validity for falls
	WHOQOL-BREF	Free	Reliability α >0.80; cross-cultural validity
	QoL-Q	Free with permission	Acceptable reliability; validity in intellectual disability
	QOLI	Commercial	Adequate reliability; validity in quality of life

	ComQOL	Free with permission	Reliability α >0.70; multidimensional validity
	SF-36 / SF-12 v.2	Free with permission	Reliability α >0.85; strong validity
	Novel QOL1/QOL2	Free	Preliminary validity in autism
	FLZ	Commercial	Acceptable reliability; validity in life satisfaction
	ASQoL	Free with permission	Reliability α >0.80; validity in autism-specific quality of life
